# A rapid visualization method for detecting rotavirus A by combining nuclear acid sequence-based amplification with the CRISPR-Cas12a assay

**DOI:** 10.1099/jmm.0.001892

**Published:** 2024-10-03

**Authors:** Yue Chen, Junhua Wu, E-bin Gao, Yanbo Lu, Haiyan Qiu

**Affiliations:** 1 Health Science Center, Ningbo University, Ningbo, Zhejiang 315000, PR China; 2 Department of Pediatrics, The Affiliated Women and Children’s Hospital of Ningbo University, Ningbo, Zhejiang 315000, PR China; 3 School of Life Sciences, Jiangsu University, Zhenjiang, Jiangsu 212000, PR China

**Keywords:** CRISPR-Cas12a, NASBA, nucleic acid detection, rotavirus

## Abstract

**Introduction.** Rotavirus A is the most common pathogen causing diarrhoea in children less than 5 years, leading to severe complications such as dehydration, electrolyte imbalances, acidosis, myocarditis, convulsions, pneumonia, and other life-threatening conditions.

**Gap statement.** There is an urgent need for a rapid and efficient nucleic acid detection strategy to enable early diagnosis and treatment, preventing rotavirus transmission and associated complications.

**Aim.** This article aimed to develop a nuclear acid sequence-based amplification (NASBA)-Cas12a system for detecting rotavirus A using fluorescence intensity or lateral flow strips.

**Methodology.** The NASBA technology was combined with the clustered regularly interspaced short palindromic repeats-Cas12a system to establish a NASBA-Cas12a system for detecting rotavirus A.

**Results.** The NASBA-Cas12a system could detect rotavirus A at 37 ℃ within 70 min and had no cross-reactivity with other viruses, achieving a limit of detection of 1.2 copies μl^–1^. This system demonstrated a sensitivity of 100%, specificity of 90%, positive predictive value of 97.22% and negative predictive value of 100%. The kappa value was 0.933, indicating that the NASBA-Cas12a system was highly consistent with reverse transcription-PCR.

**Conclusion.** The NASBA-Cas12a system exhibited high sensitivity and specificity for detecting rotavirus A, showing great potential for clinical application.

## Data Availability

The datasets generated and/or analysed during the current study are available from the corresponding author on reasonable request.

## Introduction

Rotavirus A is the main pathogen causing diarrhoea in infants and young children worldwide [1,2]. Approximately 60% of patients with acute diarrhoea worldwide are hospitalized due to rotavirus infection [3]. It occurs seasonally in autumn and winter and can cause symptoms such as fever, nausea, vomiting and diarrhoea in affected children. It can also lead to complications such as dehydration, electrolyte imbalance, acidosis, myocarditis, convulsions and pneumonia, which can be life-threatening [4]. Accurate and rapid diagnosis of rotavirus infection is crucial for determining appropriate treatment methods and preventing unnecessary antibiotic use and transmission of infection [5,6].

**Fig. 1.**
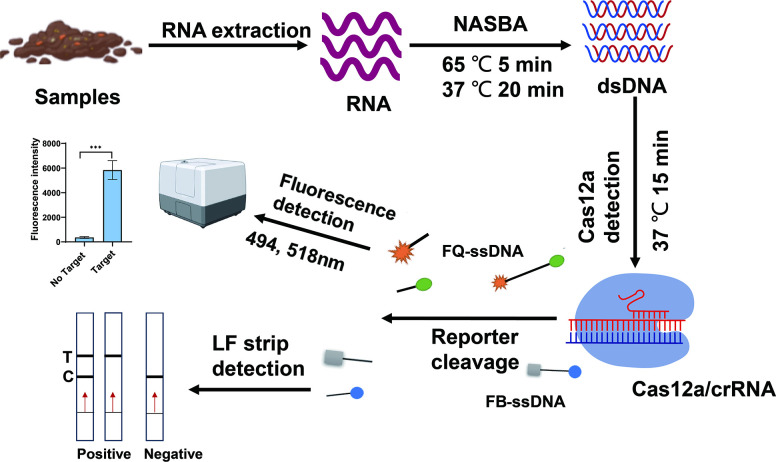
Schematic diagram of the NASBA-Cas12a system for the detection of rotavirus A. The RNA was extracted from the samples and unchained into ssRNA at 65 ℃ for 5 min. The unchained ssRNA amplified into dsDNA using NASBA. When the Cas12a/crRNA complex was bound to the complementary dsDNA, the Cas12a endonuclease was activated to cleave the target DNA and non-specific ssDNA. The FAM-BHQ ssDNA (FQ-ssDNA) reporter with a fluorophore at the 5′-end and a dark quencher at the 3′-end in the system was trans-cleaved to emit strong fluorescence. Similarly, the FAM-Biotin ssDNA (FB-ssDNA) reporter was cleaved for visualization on the LF strips.

The conventional methods of rotavirus detection, such as electron microscopy and culture, are labour-intensive, time-consuming and rarely used in clinical settings [7,8]. Currently, the most widely used methods are reverse transcription-PCR (RT-PCR) and immunological assays [6,9]. Although RT-PCR is the gold standard for diagnosis, its long operation time and requirement for highly purified samples, specialized instruments and trained professionals limit its clinical application [10]. Colloidal gold immunochromatography is a convenient and cost-effective method for detecting rotavirus. However, it may have low sensitivity at low concentrations of the virus, which may result in negative results [11]. Therefore, there is an urgent need for a rapid, convenient and sensitive detection method to improve the detection rate of rotavirus and facilitate clinical management. The discovery of the clustered regularly interspaced short palindromic repeats (CRISPR)-Cas system provides the possibility for rapid and sensitive molecular diagnosis [12]. It is an acquired immune system formed by bacteria and archaea against foreign nucleic acid attacks such as plasmids and bacteriophages, which can bind and cleave exogenous nucleic acids [13,14]. Among them, CRISPR-Cas12a is an RNA-guided endonuclease that can cleave target DNA and selectively cleave any ssDNA [15]. These effector proteins are useful tools for genome editing and gene regulation applications due to the variability of crRNA sequences [13,16]. To determine the presence of the target nucleic acid, a labelled ssDNA is designed, and its cleavage indicates the presence of the target. Currently, the CRISPR-Cas12a system has been utilized for the detection of COVID-19, norovirus, hepatitis B virus and influenza A and B viruses [10,17,22]. To enhance detection sensitivity, it is often combined with isothermal amplification technologies to amplify the reaction, such as loop-mediated isothermal amplification (LAMP), recombinase polymerase amplification (RPA), rolling circle amplification (RCA), helicase-dependent amplification and nuclear acid sequence-based amplification (NASBA) [23,26]. Among these techniques, NASBA is a transcription-based isothermal amplification method that uses RNA as a template [27]. Its amplification products include ssRNA, dsDNA and ssDNA. The entire reaction does not require thermal cycling instruments and has high sensitivity and specificity [28]. In this study, we developed a NASBA-Cas12a system for the detection of rotavirus A, which combined the NASBA and Cas12a/crRNA assays using fluorescence intensity or lateral flow (LF) strips (Fig. 1). The NASBA-Cas12a system showed rapid, high sensitivity and specificity for detecting rotavirus A under constant temperature conditions, making it suitable for clinical applications.

## Methods

### Materials and reagents

Faecal samples were collected from children suspected of being clinically diagnosed with rotavirus infection, as well as from healthy children, at the Affiliated Women and Children’s Hospital of Ningbo University. These samples were promptly stored at −80 °C for preservation. All participants obtained parental consent and signed informed consent. The study was reviewed by the Ethics Committee of the Affiliated Women and Children’s Hospital of Ningbo University and adhered to the tenets of the Declaration of Helsinki.

Primers and ssDNA reporters were all synthesized by Sangon Biotech (Shanghai, China). Recombinant LbCRISPR-Cas12a protein was purchased from HuiCheng Biotechnology (Shanghai, China). T7 RNA polymerase and RNase H were purchased from Thermo Fisher Scientific Inc. (USA). Reverse transcriptase and RNase Inhibitor were purchased from Vazyme Biotechnology (Nanjing, China). LF strips were purchased from Xianda Biotechnology (Suzhou, China). The virus RNA Extraction Kit was purchased from TIANGEN (Beijing, China).

### Target DNA and crRNA preparation

The conserved sequence of the rotavirus VP6 gene was synthesized and ligated into a plasmid pUC57 (Sangon, Shanghai). Afterwards, the target sequence was used to design the crRNAs and primers of NASBA.

For the preparation of crRNAs, three TTTN-containing sequences (crRNA1, crRNA 2 and crRNA3) were designed based on target DNA. The transcription template dsDNA was prepared by amplifying plasmid DNA with the primers of T7-crRNA-F and gRNA (1,2,3). Then, 1 μg PCR products were transcribed at 37 ℃ for 4 h using the T7 transcription kit and purified with the 5-min purification kit (Hai Gene, Harbin, China). Finally, the crRNAs were quantified using NanoDrop 2000C and stored at −80 ℃.

### NASBA assays

To improve the sensitivity of the assay, the target RNA was amplified using the NASBA method. The NASBA reaction system contained 40 mM Tris-HCl (pH 8.5), 12 mM MgCl_2_, 50 mM KCl, 5 mM DTT, 15% DMSO, 1 mM dNTP, 2 mM NTP, 0.5 U RNase H, 40 U T7RNA polymerase, 20 U M-MuLV reverse transcriptase, 10 U RNase inhibitor, 0.2 µM primer RV-PF/RV-PR and RNA template 2 µl and filled to 25 µl with RNase-free water. The results were shown by 2% agarose gel electrophoresis to determine the appropriate amplification product for subsequent experiments.

### Cas12a/crRNA-activated Cis-cleavage assay

The Cas12a/crRNA assay for target and reporter cleavage was performed in a 20 µL mixture containing 2 µL of 10× cleavage buffer (600 mM NaCl, 60 mM MgCl_2_ and 500 mM Tris-HCl pH 7.5), 125 nM of Cas12a, 500 nM of FQ1-ssDNA reporter, 500 nM of crRNA, 2 µL of target amplicons and sterile nuclease-free water. The mixture was incubated at 37 °C for 60 min to ensure sufficient cleavage. The trans-cleavage for fluorescence detection was monitored using a fluorescence enzyme-linked immunosorbent assay instrument Synergy H1 (λex, 494 nm; λem, 518 nm).

To determine the optimal Cas12a-mediated trans-cleavage activity, three crRNA sequences (crRNA1, crRNA2 and crRNA3) and four FAM-BHQ ssDNA (FQ-ssDNA) (FQ1, FQ2, FQ3 and FQ4) were designed to screen for the optimal crRNA and FQ-ssDNA. The ratio of Cas12a to crRNA was set to 4 : 1, 2 : 1, 1 : 1, 1 : 2, 1 : 4 and 1 : 8. The reaction time was set from 10 to 60 min, and the temperature was set at 25 ℃, 30 ℃, 37 ℃, 40 ℃ and 45 ℃. The reaction conditions were as described above.

### The specificity and sensitivity of the Cas12a/crRNA assay

The specificity and sensitivity of the Cas12a/crRNA assay were confirmed using fluorescence intensity and LF strips. For the LF strips, the Cas12a/crRNA assay was performed as described above, except that the FQ-ssDNA reporter was replaced by the FAM-Biotin ssDNA (FB-ssDNA) reporter. The reaction products were diluted to 50 µl, and LF strips were then immersed in the resulting reactions. A C-line, close to the sample application pad, indicated a negative result, whereas a T line close to the top of the strip or two bands indicated a positive result.

To verify the specificity of the Cas12a/crRNA assay, a total of four groups of DNA genomes were selected from norovirus (NoV), influenza A virus (IAV), influenza B virus (IBV) and adenovirus (ADV) and compared with rotavirus A. The limit of detection (LOD) was determined in triplicate using the following tenfold dilution series of DNA products ranging from 5×10^10^ to 5×10^5^ copies μl^–1^ as templates for the Cas12a/crRNA assay.

### The evaluation of the NASBA-Cas12a system

The LOD of the NASBA-Cas12a system was determined by using the following tenfold dilution series of RNA ranging from 1.2×10^5^ to 1.2×10^−1^ copies μl^–1^ as templates for the NASBA assay, and the NASBA amplified products were used in the subsequent Cas12a/crRNA fluorescence detection and LF strip detection. Meanwhile, reverse transcription-quantitative PCR (RT-qPCR) was performed on 10-fold dilution series of RNA to determine its LOD. We collected ten faecal samples from healthy children who were confirmed negative for rotavirus by RT-qPCR. RNA was extracted from these samples. We prepared a solution by mixing the RNA extracted from these negative samples with the standard RNA of rotavirus in a 1 : 1 ratio. This mixture was then subjected to tenfold serial dilutions followed by NASBA amplification and detection using the Cas12a/crRNA assay with fluorescence detection and LF strip detection.

### Clinical sample analysis

To further validate the feasibility of the NASBA-Cas12a system in clinical samples, faecal samples from 45 children with clinical suspicion of rotavirus infection were collected in this study. Extracting RNA from faecal samples involved suspending 1 ml of faeces in 5 ml of normal saline, vortexing thoroughly and centrifuging at 4000 ***g*** for 20 min to remove faecal impurities, resulting in 140 µl of supernatant. The RNA extraction used the TIANGEN virus RNA extraction kit, which employed centrifugal adsorption columns to efficiently and specifically adsorb RNA, removing protein impurities and other contaminants. Finally, the RNA was eluted with 60 µl of RNase-free H_2_O, using NanoDrop to quantify the RNA and to ensure the quality of purified RNA. The samples were stored at −80 °C for subsequent detection. Total RNA was reacted at 65 ℃ for 5 min to unchain the dsRNA into ssRNA. The RT-qPCR was performed using HiScript III first Strand cDNA Synthesis Kit (+gDNA wiper), followed by q-PCR with the primers of RV-F and RV-R. The ssRNA was detected by parallel detection methods of the NASBA-Cas12a system and RT-qPCR. To protect the privacy of the children, the collected samples were re-numbered. All samples were repeated three times, and the detection results of the two methods were calculated. Based on the results of RT-qPCR, the detection efficiency of NASBA-Cas12a system was analysed.

### Data statistical analysis

Statistical analyses were performed using GraphPad Prism 9.5, with differences between two or more groups compared using independent-samples *t*-tests or one-way ANOVA. All experiments were repeated at least three times, and data are shown as mean±sd. Differences were considered statistically significant when the *P*-value was <0.05. The signal-to-noise ratio (S/N) is equal to the end fluorescence intensity of the experimental group/negative control group. In RT-qPCR reaction, Ct value ≥35 can be considered a negative result. The consistency of the two detection methods was determined by the kappa test, kappa ≥0.8, which was considered to have good consistency.

## Results

### Establishment of the Cas12a/crRNA assay

**Fig. 2.**
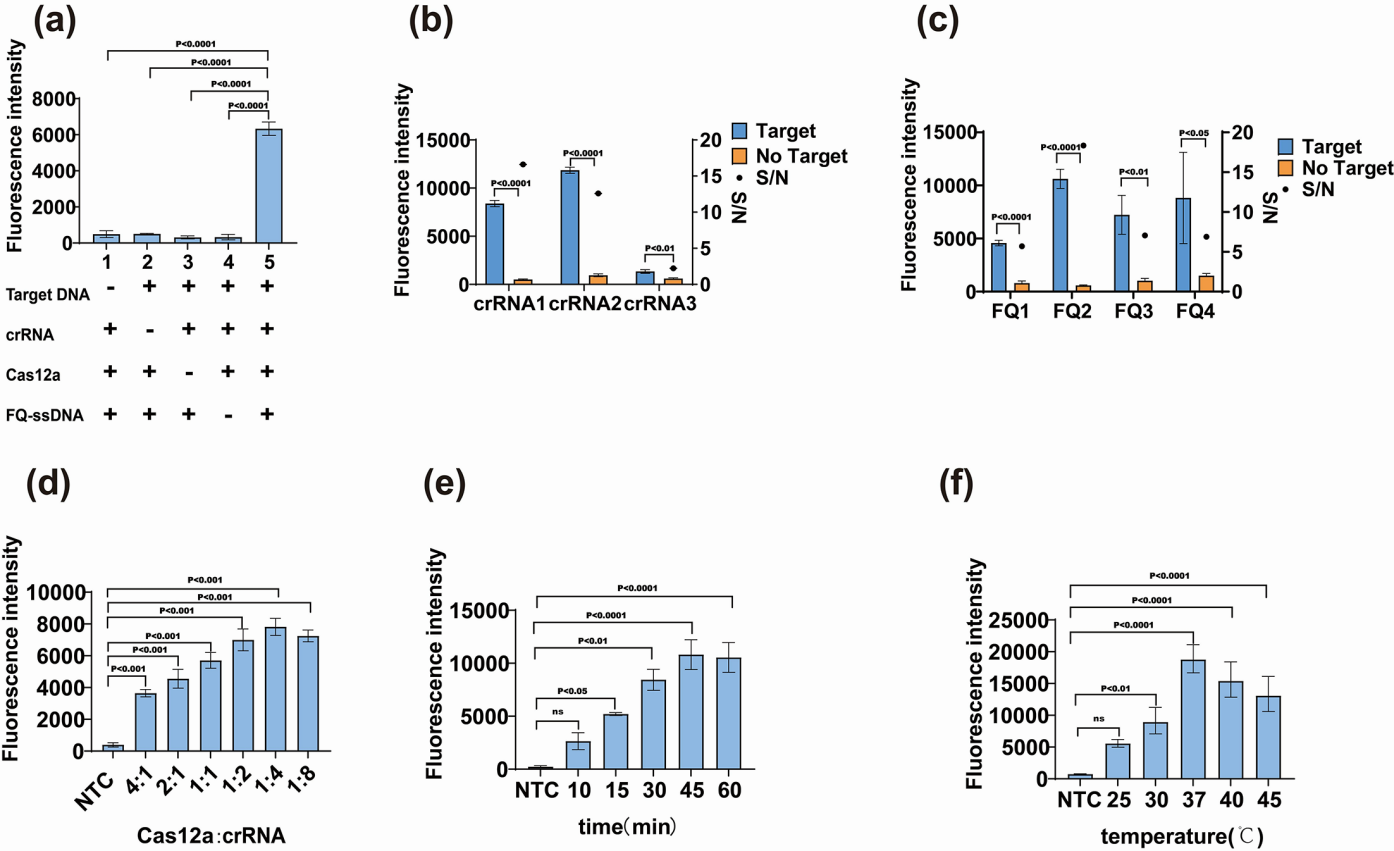
Feasibility analysis and optimization of the Cas12a/crRNA assay for the detection of rotavirus A. (a) Evaluation of five reactions with different components by endpoint fluorescence detection. (b) Analysis of fluorescence results triggered by different crRNAs. (c) Analysis of fluorescence results triggered by different FQ-ssDNA. (d) Fluorescence results of the Cas12a/crRNA assay with different Cas12a:crRNA ratios. (e) Fluorescence results of the Cas12a/crRNA assay with different reaction times. (f) Fluorescence results of the Cas12a/crRNA assay with different reaction temperatures. All parameters were evaluated by endpoint fluorescence intensity. Data are expressed as mean±sd of the difference between the means of three technical replicates. NTC, no target control; S/N, ratio of target to no target fluorescence intensity; ns, no statistical difference.

We screened out the conservative sequence of rotavirus and synthesized the plasmids as a positive template for subsequent experimental design. Based on this target region, the crRNA and amplification primers were designed and shown in Table 1. To systematically evaluate the Cas12a/crRNA assay, we prepared and tested five reaction assays (reactions 1–5) with different components. After incubation at 37 °C for 60 min, only reaction 5 containing target DNA, crRNA, Cas12a and FQ-ssDNA produced a super-bright fluorescence signal (Fig. 2a). The results of fluorescence showed that the presence of all components of the assay could activate the trans-cleavage activity of Cas12a/crRNA, and the FQ-DNA reporter was cleaved and released a fluorescent signal.

**Table 1.** The sequences of primers, crRNA, FQ-ssDNA and target DNA used in this study

Name	Sequence (5′−3′)
T7-crRNA-F	GATCACTAATACGACTCACTATAGGGTAATTTC
gRNA1	TTGTGAATCAGTGCTTGCGGATGATCTACACTTAGTAGAAATTACCCTATAGTGAGTCG
gRNA2	CTCAGTCCAGTTCATGCCTGGTGATCTACACTTAGTAGAAATTACCCTATAGTGAGTCG
gRNA3	GAGGCTACTGTAAAGACACGTTGATCTACACTTAGTAGAAATTACCCTATAGTGAGTCG
RV-PF	AACATCATGCWACRGTWGGACT
RV-PR	AATTCTAATACGACTCACTATAGGGAGAGAGAGAGCAGATGGTTAGYYTGGTCCTYA
FQ2	6FAM-CGATCGAGTCAAATCCAGCYACTTGACGATCG-BHQ1
FQ1	6FAM-TTATTATT-BHQ1
FQ3	6FAM-GGTTCGTAGAGCACACGAATAGCGAACC-BHQ1
FQ4	6FAM-CCAAGCAACTCAGGAAACARGGTGTCGCTTGG-BHQ1
crRNA1	UAAUUUCUACUAAGUGUAGAUCAUCCGCAAGCACUGAUUCACAA
crRNA2	CUCAGUCCAGUUCAUGCCUGGUGAUCUACACUUAGUAGAAAUUA
crRNA3	GAGGCUACUGUAAAGACACGUUGAUCUACACUUAGUAGAAAUUA
RV-F	CCGCAGTACGTCAAGAATATGC
RV-R	GCAAATTATCTTCCCTAGATGGTGAA
VP6	TCAACATCATGCAACAGTTGGACTTACGTTACGTATTGAGTCTGCAGTTTGTGAATCAGT GCTTGCGGATGCAAATGAAACTTTATTGGCAAATGTTACCGCAGTACGTCAAGAATATG CTATACCAGTTGGACCAGTATTTCCACCAGGCATGAACTGGACTGAGCTGATTACTAACT ATTCACCATCTAGGGAAGATAATTTGCAACGTGTCTTTACAGTAGCCTCTATCAGAAGCA TGTTGATTAAGTGAGGACCAGACTAACCATCTGGTATCCAATCTTAGGTATCCAATCTTAG

Since the efficiency of each crRNA in triggering the trans-cleavage ability of Cas12a may be different, we designed and screened the optimal one among the designed crRNAs. The fluorescence detection results showed that all of the crRNAs could bind to Cas12a for cleavage reaction (*P*<0.05). While crRNA2 showed the highest fluorescence intensity with a low S/N, crRNA1 showed higher fluorescence intensity and the highest S/N (Fig. 2b), so crRNA1 was selected to be combined with Cas12a to perform the target cleavage reaction. To select a suitable ssDNA reporter for the experiment, four groups of FQ-ssDNA were selected for Cas12a reaction. As shown in Fig. 2c, all FQ-ssDNA reporters were cleaved successfully, and FQ2 generated stronger fluorescence intensity compared to the others. Therefore, the FQ2 was selected as the optimal signal reporter and was used in the subsequent Cas12a cleavage experiments.

To improve the efficiency of the Cas12a/crRNA assay, the Cas12a cleavage parameters were optimized, including the ratio of Cas12a to crRNA, reaction time and temperature, using a constant concentration of the rotavirus A VP6 gene. We evaluated the ratio of Cas12a to crRNA by fixing Cas12a at 125 nM and adjusting the concentration of crRNA to achieve ratios of 4 : 1, 2 : 1, 1 : 1, 1 : 2, 1 : 4 and 1 : 8. The results showed that the lower the ratio of Cas12a to crRNA, the stronger the fluorescence intensity, and the maximum fluorescence intensity observed at the ratio of 1 : 4 (Fig. 2d). Therefore, the ratio of Cas12a to crRNA was chosen to be 1 : 4 for the following experiments. As for the reaction time, 10, 15, 30, 45 and 60 min were tested; the results showed that there was a statistical difference between the target group and no-target group at 15 min of reaction (*P*<0.05), with the fluorescence intensity reaching a plateau after 45 min (Fig. 2e). For the optimal temperature of the reaction, 25 ℃, 30 ℃, 37 ℃, 40 ℃ and 45 ℃ were tested, with 37 °C identified as the optimal temperature (Fig. 2f). Therefore, the optimized conditions for the Cas12a/crRNA assay to detect rotavirus A were as follows: a Cas12a-to-crRNA ratio of 1 : 4, a reaction time of 15 min and an optimal temperature of 37 °C.

### Specificity and sensitivity of the Cas12a/crRNA assay

**Fig. 3.**
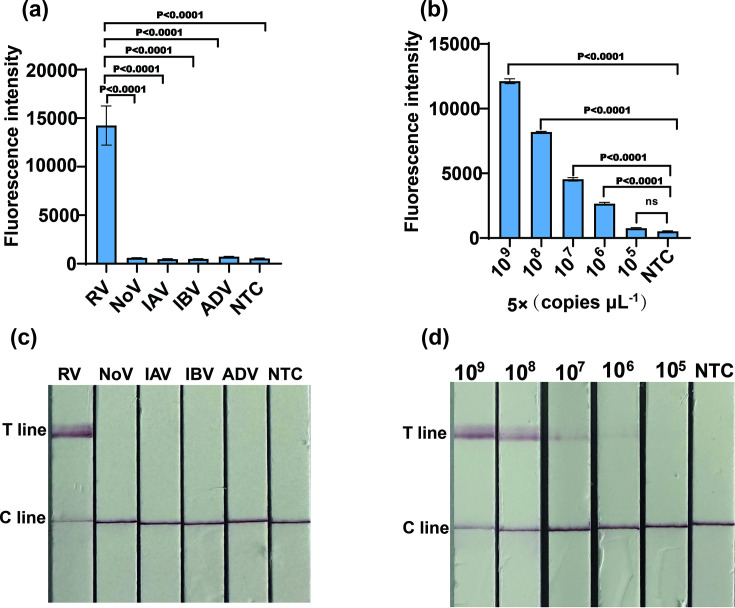
Specificity and sensitivity of the Cas12a/crRNA assay for the detection of rotavirus A. (**a**) Specificity of the fluorescence for the Cas12a/crRNA assay. (**b**) Sensitivity of the fluorescence for the Cas12a/crRNA assay. (**c**) Specificity of LF strips for the Cas12a/crRNA assay. (**d**) Sensitivity of the LF strips for the Cas12a/crRNA assay.

The specificity of the Cas12a/crRNA assay was evaluated using a standard DNA template. The genes of rotavirus A and other different RNA virus genes (NoV, IAV, IBV and ADV) were used to assess the specificity of the Cas12a/crRNA assay. As demonstrated in Fig. 3a, the fluorescence intensity could be detected in rotavirus, but not in the strains of other viruses, and only the RV group had the T line in the LF strips, indicating no cross-reaction of the Cas12a/crRNA assay in the detection of rotavirus (Fig. 3c). Therefore, the assay showed high specificity for rotavirus.

To evaluate the sensitivity of the Cas12a/crRNA assay, a serial dilution of DNA standard template from 5×10^9^ to 5×10^5^ copies μl^–1^ was detected in the cleavage assay. The results showed that the fluorescence intensity was gradually decreased, and the T line was gradually shallower until it disappeared at 5×10^5^ copies μl^–1^, indicating that the LOD mediated by this assay was 5×10^6^ copies μl^–1^ (Fig. 3b, d).

### Sensitivity of NASBA-Cas12a system

**Fig. 4.**
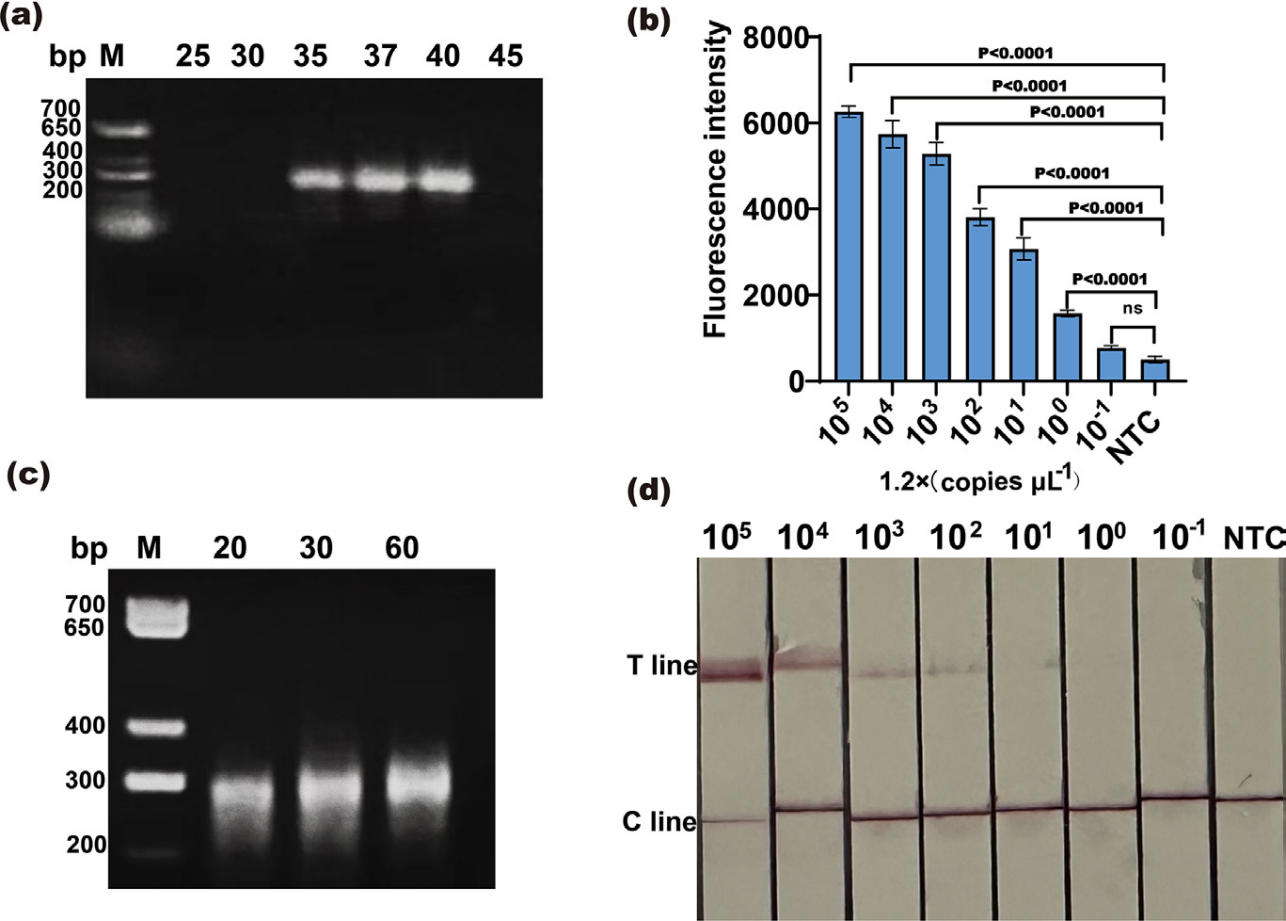
Construction of the NASBA-Cas12a system. (**a**) Gel electrophoresis analysis of NASBA products amplified at different temperatures. (**b**) Analytical sensitivity of the fluorescence for the NASBA-Cas12a system. (**c**) Gel electrophoresis analysis of NASBA products amplified at different time points. (**d**) Analytical sensitivity of the LF strips for the NASBA-Cas12a system.

To improve the sensitivity of the Cas12a/crRNA assay in the detection of rotavirus A, the NASBA was performed to facilitate the development of the highly sensitive detection. Since temperature and time are critical factors in the NASBA product detection, we initially optimized the reaction temperature, ranging from 25 ℃ to 45 ℃. The results showed that the target band could be amplified at temperatures between 35 °C and 40 °C (Fig. 4a). To ensure the consistency of the reaction temperature between the NASBA and Cas12a/crRNA assays, we considered 37 °C as the reaction temperature for NASBA. Similarly, to select the appropriate amplification time, we set the reaction time of 20, 30 and 60 min. The results showed that a clear band appeared at 20 min (Fig. 4c). Therefore, the reaction temperature of 37 ℃ and the reaction time of 20 min were selected to perform the NASBA assay.

To determine the sensitivity of the NASBA-Cas12a system, the target RNA was serially diluted from 1.2×10^5^ to 1.2×10^−1^ copies μl^–1^, 2 µl of RNA was used as a template in NASBA, and 2 µl of the amplified products were then detected with the Cas12a/crRNA assay. The results showed that the fluorescence intensity gradually decreased up to 1.2×10^−1^ copies μl^–1^ that there was no difference with NTC, and the T line of the LF strips gradually became lighter until it disappeared at 1.2×10^−1^ copies μl^–1^ (Fig. 4b and d). RT-qPCR was performed on tenfold dilution series of RNA, and the results showed that the LOD of RT-qPCR was 12 copies μl^–1^ (Fig. S1, available in the online Supplementary Material). Additionally, a solution was prepared by mixing RNA-negative rotavirus solution with standard RNA in a 1 : 1 ratio. This RNA solution was then subjected to tenfold serial dilutions followed by NASBA amplification and detection using the Cas12a/crRNA assay with fluorescence and LF strips assays. The results showed that nine groups had fluorescence and LF strip detection results at a single copy, while one group had an LOD below a single copy (Fig. S2). These results illustrated that the LOD of our NASBA-cas12a assay was a single copy.

### Clinical sample analysis

**Fig. 5.**
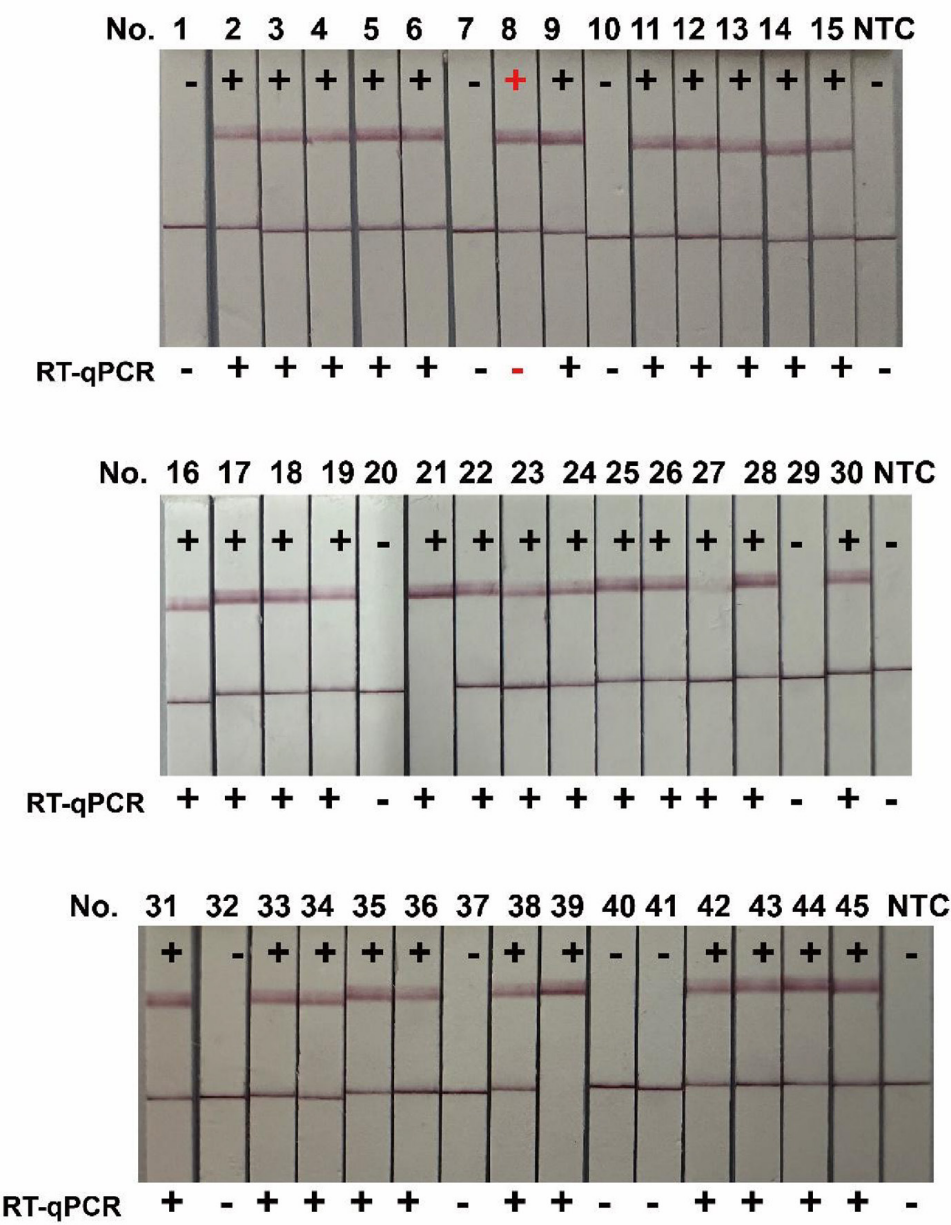
Comparison of the NASBA-Cas12a LF strip system and RT-qPCR detection results.

To validate the reliability of the NASBA-Cas12a system, we collected 45 clinical faecal samples suspected of rotavirus infection. Parallel testing was conducted using both the NASBA-Cas12a system and the RT-PCR gold standard. According to RT-qPCR, 35 samples were positive, and 10 samples were negative. For the NASBA-Cas12a system, LF strips were used to determine the results. Samples were considered positive if they showed only the T line or both the T and C lines and negative if only the C line appeared. The final results showed 36 positive and 9 negative results with the NASBA-Cas12a system. Only sample number 8 had a different result (Fig. 5). The sensitivity of the NASBA-Cas12a system was 100%, and the specificity was 90%. The positive predictive value (PPV) relative to RT-qPCR was 97.22%, and the negative predictive value (NPV) was 100%. The kappa value calculated was 0.933 (Table 2), indicating a high level of agreement between the two methods for detecting rotavirus A.

**Table 2.** Concordance analysis between the NASBA-Cas12a system and RT-qPCR

		RT-qPCR		Total
		Positive	Negative	
NASBA-Cas12a	Positive	35	1	36 (PPV: 97.22%)
	Negative	0	9	9 (NPV: 100%)
Total		35	10	45

## Discussion

Rotavirus A is a non-enveloped, dsRNA virus belonging to the genus *Rotavirus* and family *Reoviridae* [29]. It is one of the main pathogens causing diarrhoea in children. It poses a serious threat to children’s lives and health, and rapid diagnosis is crucial for treatment.

In this study, we developed a rapid detection method for rotavirus A using the NASBA-Cas12a system. We identified the VP6 sequence as a highly conserved sequence for rotavirus A and screened the optimal crRNA and FQ-ssDNA reporter in the Cas12a/crRNA assay. In addition, we also optimized the reaction conditions of the assay. The NASBA-Cas12a system was established by integrating NASBA with the Cas12a/crRNA assay, using LF strips for visual detection, and the LOD reached 1.2 copies μl^–1^. Moreover, it had high specificity, which did not have cross-reactivity with other common virus infections in children. Validation of clinical samples showed that this method had high consistency with RT-qPCR.

Currently, researchers often combine isothermal amplification with the CRISPR-Cas12a assay to reduce the LOD, such as RPA, LAMP and RCA. They were all based on DNA as the template. For RNA viruses, such as rotavirus A, which require RNA as the target template for Cas12a detection, an additional reverse transcription step is traditionally necessary, adding complexity to the detection process [30,32]. We used the NASBA to amplify RNA targets, omitting the reverse transcription step. Our system offered several advantages. Both the NASBA and the Cas12a/crRNA assays were performed at 37 ℃, eliminating the potential influence of temperature variations on the detection results. NASBA selectively amplifies template nucleic acid with a T7 promoter, which avoids potential interference from non-target nucleic acids, thus enhancing detection specificity [33], and the NASBA amplification products can be directly used for the subsequent Cas12a/crRNA assay without purification or other operations, simplifying the experimental procedure and saving time. The system utilized LF strips for direct result observation, reducing the reliance on large, complex instruments. The entire process can be completed with only a small centrifuge and a water bath, eliminating the need for sophisticated equipment or highly skilled personnel. The NASBA-Cas12a system had high specificity and sensitivity and showed high consistency with the gold standard RT-PCR.

Undeniably, this experiment had certain limitations. This method for detecting rotavirus still requires the use of a centrifuge for RNA extraction, which presents some limitations for on-site detection. We also need to perform NASBA amplification of the RNA. After amplification, the cap must be opened to carry out Cas12a/crRNA detection, which requires at least two steps to complete the experiment. Opening the cap during this process could increase the risk of aerosol contamination, leading to potential false-positive results. The fragments and other factors in faecal samples that may affect LF strip results and the reagents, kits and LF strips required for our current experiments are still relatively expensive. Our future work involves simplifying the reaction process, developing integrated reagent kits to reduce costs and improving the material and structure of the LF strips to enhance the anti-interference ability and detection efficiency. Through the detection of a large number of faecal samples, various parameters of the LF strips can be adjusted and optimized to ensure effectiveness in complex samples. Multi-centre validation in different laboratories and environments is critical to validate the stability and repeatability of the method. We aim to utilize microfluidic chip detection methods that do not require professional technical personnel or complex equipment. The process will only require sample addition to complete the detection, ensuring that it is contamination-free and promotes accurate result interpretation. This will enhance detection efficiency, benefiting both clinical and on-site testing applications.

In conclusion, the development of the NASBA-Cas12a system provides a rapid, highly specific and highly sensitive method for the detection of rotavirus A while also offering an alternative detection method for other RNA viruses. We hope this method can provide effective support for the prevention and treatment of rotavirus infections and offer inspiration and reference for the detection of other RNA viruses.

## supplementary material

10.1099/jmm.0.001892 Uncited Fig. S1.

## Abbreviations

ADV: adenovirus
CRISPR: clustered regularly interspaced short palindromic repeats
dsDNA: double-stranded DNA
dsRNA: double-stranded RNA
FB-ssDNA: FAM-Biotin ssDNA
FQ-ssDNA: FAM-BHQ1 ssDNA
IAV: influenza A virus
IBV: influenza B virus
LAMP: loop-mediated isothermal amplification
LF: lateral flow
LOD: limit of detection
NASBA: nucleic acid sequence-based amplification
NoV: norovirus
NPV: negative predictive value
PPV: positive predictive value
RCA: rolling circle amplification
RPA: recombinase polymerase amplification
RT-PCR: reverse transcription-PCR
S/N: signal-to-noise ratio
ssDNA: single-stranded DNA
ssRNA: single-stranded RNA
