# Supplementary material for: A rapid visualization method for detecting rotavirus A by combining nuclear acid sequence-based amplification with the CRISPR-Cas12a assay
Source: J Med Microbiol. 2024 Oct 3;73(10):001892. doi: 10.1099/jmm.0.001892 (PMC11448473; doi:10.1099/jmm.0.001892)
Supplement: Uncited Fig. S1. [file jmm-73-01892-s001.pdf]

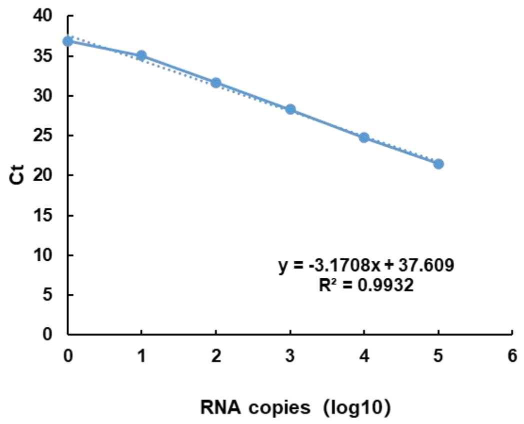

Fig. S1. Linear relationship between standard RNA copy number and Ct value.

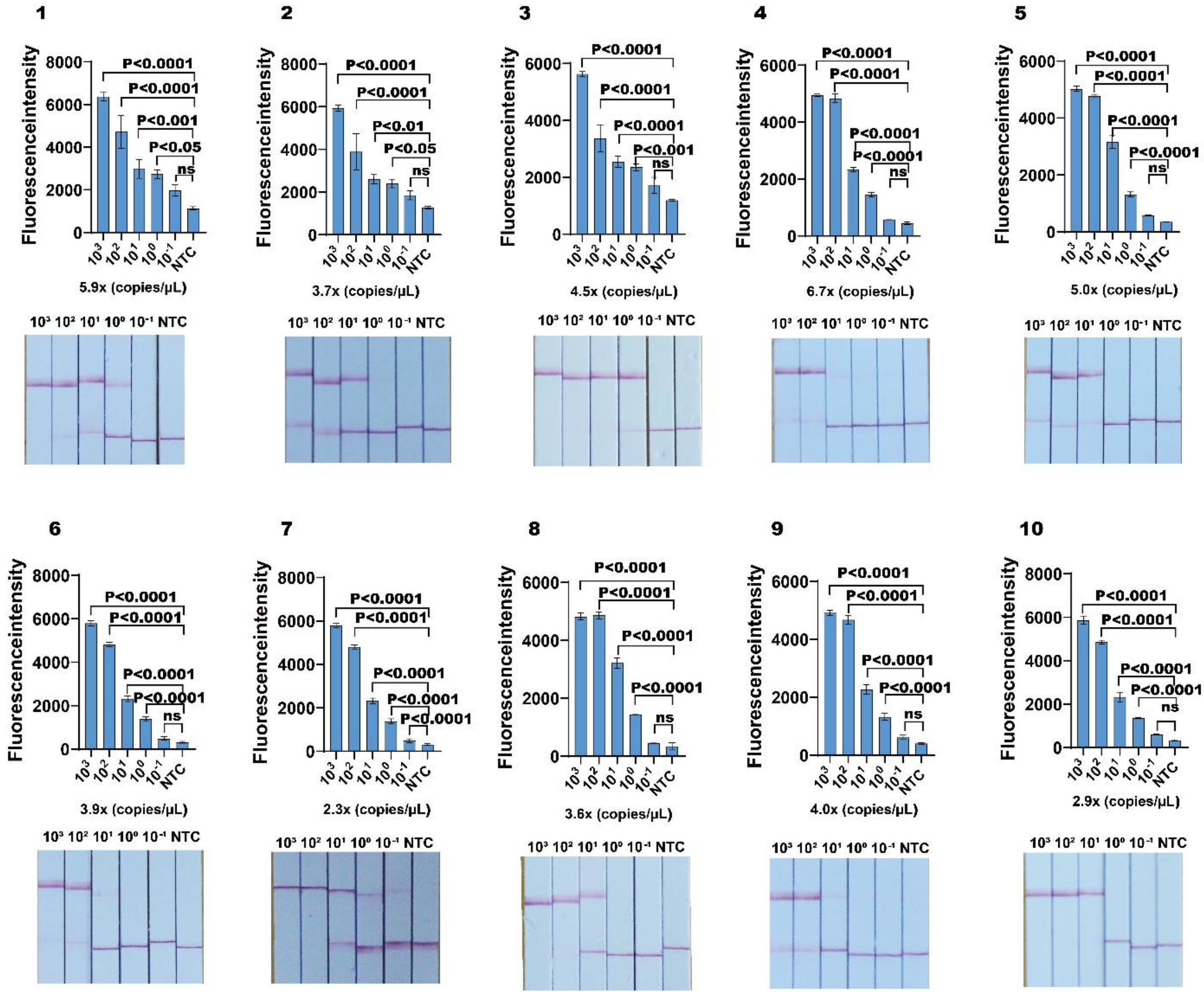

Fig. S2. 10 groups of negative fecal RNA samples were mixed with standard RNA for 10-fold dilution of NASBA-Cas12a fluorescence and test strip detection.
